# Viral load of SARS-CoV-2 in droplets and bioaerosols directly captured during breathing, speaking and coughing

**DOI:** 10.1038/s41598-022-07301-5

**Published:** 2022-03-03

**Authors:** Tyler J. Johnson, Robert T. Nishida, Ashlesha P. Sonpar, Yi-Chan James Lin, Kimberley A. Watson, Stephanie W. Smith, John M. Conly, David H. Evans, Jason S. Olfert

**Affiliations:** 1 Department of Engineering, University of Cambridge, Cambridge, UK; 2 Department of Mechanical Engineering, University of Alberta, Edmonton, Canada; 3 Department of Medicine, Division of Infectious Diseases, University of Alberta, Edmonton, Canada; 4 Alberta Health Services, Alberta, Canada; 5 Department of Medical Microbiology and Immunology and Li Ka Shing Institute of Virology, University of Alberta, Edmonton, Canada; 6 Department of Family Medicine, University of Alberta, Edmonton, Canada; 7 Department of Medicine, Microbiology, Immunology and Infectious Diseases, Pathology and Laboratory Medicine, Synder Institute for Chronic Diseases and O’Brien Institute for Public Health, Cumming School of Medicine, University of Calgary, Calgary, Canada

**Keywords:** Viral infection, Infectious diseases

## Abstract

Determining the viral load and infectivity of SARS-CoV-2 in macroscopic respiratory droplets, bioaerosols, and other bodily fluids and secretions is important for identifying transmission modes, assessing risks and informing public health guidelines. Here we show that viral load of SARS-CoV-2 Ribonucleic Acid (RNA) in participants’ naso-pharyngeal (NP) swabs positively correlated with RNA viral load they emitted in both droplets >10 $\upmu \hbox {m}$ and bioaerosols <10 $\upmu \hbox {m}$ directly captured during the combined expiratory activities of breathing, speaking and coughing using a standardized protocol, although the NP swabs had $\approx$ 10$^3\times$ more RNA on average. By identifying highly-infectious individuals (maximum of 18,000 PFU/mL in NP), we retrieved higher numbers of SARS-CoV-2 RNA gene copies in bioaerosol samples (maximum of 4.8${\times }10^{5}$ gene copies/mL and minimum cycle threshold of 26.2) relative to other studies. However, all attempts to identify infectious virus in size-segregated droplets and bioaerosols were negative by plaque assay (0 of 58). This outcome is partly attributed to the insufficient amount of viral material in each sample (as indicated by SARS-CoV-2 gene copies) or may indicate no infectious virus was present in such samples, although other possible factors are identified.

## Introduction

The COVID-19 pandemic is caused by transmission of SARS-CoV-2 from person-to-person. SARS-CoV-2 can be transmitted through droplets, aerosols or contact (direct or indirect via fomites) from an infected person through saliva and respiratory secretions or other body fluid secretions containing SARS-CoV-2. These secretions include respiratory droplets larger than 5–10 $\upmu \hbox {m}$ in diameter^1,2^ which are expelled through the air during natural expiratory activities of coughing, sneezing, talking or singing^2,3^. It is known that droplets as well as droplet nuclei, which are defined as being smaller than 5 $\upmu \hbox {m}$ in diameter^1^, can be generated by breathing, speaking, coughing, singing and sneezing, and suspended in air as a distribution of particle sizes^4–9^. Furthermore, the physical behaviour and eventual fate of the particles is primarily governed by their size^10,11^. However, more evidence is needed to quantify the RNA viral load and possible infectivity of SARS-CoV-2 in emitted droplets or droplet nuclei to inform risk assessments of different possible modes of transmission^12–14^.

SARS-CoV-2 RNA has been detected by nucleic acid-based tests (NATs) such as droplet digital polymerase chain reaction (ddPCR) and quantitative reverse-transcription PCR (RT-qPCR) analyses of indoor air and surface samples in a range of (mostly clinical) settings^12^, including in droplet nuclei suspended in air^15,16^. Cycle thresholds (Ct) for RT-qPCR reported for air samples are often >35^12^ (where lower Ct corresponds to higher quantities of viral RNA) and are considered unlikely to yield cultivable virus (i.e. demonstrate infectivity of the virus ex situ)^17,18^. Of indoor air studies in which viral culture was attempted^18–27^, only limited evidence of viral replication or cytopathic effects (CPE) has been demonstrated for air samples^19,25,27^, despite in some cases sampling within 1–2 m of infected persons^20^ carrying infection-competent virus^21^. CPE on cell monolayers can also be produced by common microbial contaminants, such as bacteria and fungi, and thus is not conclusive evidence of virus infectivity. Furthermore, these previous studies did not determine the size of the pathogen-laden droplets/droplet nuclei or identify the exact source of the presumed pathogen-laden aerosol.

SARS-CoV-2 RNA has been detected by NATs in samples of air directly emitted by participants infected with COVID-19. Ma et al.^28^ detected SARS-CoV-2 RNA in 14 of 52 (26.9%) of exhaled breath condensate (EBC) samples (for N gene, RNA was detected 11 of the 52 samples and the mean Ct for those 11 samples was 35.54). These EBC samples were collected by having each of the 49 participants exhale through a long straw over 5 min, while cooling the exhaled air and and condensing the droplets into a solution^28,29^. Feng et al.^30^ gathered samples of exhaled breath using a NIOSH bioaerosol sampler (which separated particles into three ranges of particle diameters: <1 $\upmu \hbox {m}$, 1–4 $\upmu \hbox {m}$, and >4 $\upmu \hbox {m}$) from participants who were asked to breathe normally for 30 min and perform 10 forced coughs. Participants were also asked to blow into a custom EBC sampler for 10 min. In that study, SARS-CoV-2 RNA was detected in 0 of 9 exhaled breath samples and 2 of 8 EBC samples. Other recent studies also detect SARS-CoV-2 in EBC samples by RT-PCR^29,31^. Malik et al.^32^ detected a mean of 2.47${\times }10^{3}$ gene copies of SARS-CoV-2 RNA in 20 exhaled breaths (EB), which was 3–4 orders of magnitude lower than the RNA detected in the same participants’ oronasopharyngeal (ONP) swabs. The authors did not find a correlation between RNA from EB and ONP swabs^32^. Cycle thresholds as low as 29.51 (N gene) have been reported^28^ for exhaled air samples, but are typically Ct > 30 (N gene). SARS-CoV-2 RNA has also been detected by sampling masks that participants wore during natural expiratory activities. Samples were gathered by swabbing the mask^33–35^ or by testing electrostatic filters^36^, gelatin filters^37^ or polyvinyl alcohol (PVA) sampling matrix strips^38^ placed within the masks. In other studies, samples gathered 10 cm from each participant’s chin^39,40^ or by sampling EBC^41^ were negative for the presence of SARS-CoV-2 RNA by RT-PCR. It is unclear if non-aerosolized saliva (e.g. direct contact, spitting or drooling) may contaminate samples gathered directly from masks, sampling strips in masks, or EBC and therefore if they adequately represent exhalatory activities such as breathing. Of studies to date which report direct sampling of air emitted by participants infected with COVID-19, attempts to culture the virus have not been reported for either the participants’ respiratory tract fluid (e.g. NP swab) or exhaled air samples.

In this study, participants were identified by a recent positive oro-pharyngeal (OP) or naso-pharyngeal (NP) swab, and the droplets and bioaerosols they emitted were continuously sampled for ex situ virological analyses. A custom sampling apparatus was used to collect droplets and bioaerosols emitted during natural expiratory activities following a standardized protocol and determine the presence of SARS-CoV-2 and its infectivity as an approximate function of particle size by both RT-qPCR and viral culture methods.

## Results

Samples of droplets and bioaerosols emitted during combined expiratory activities (i.e. breathing, speaking and coughing following a standard procedure) and NP swabs were gathered from study participants (*n*=17; male, *n* = 8; female, *n*=9) between August 2020 and March 2021 in Calgary and Edmonton, Canada. Participants were identified by a recent positive NP swab from the community (Participants P01A–P12B; *n*=12) or hospital inpatients (P13C–P17C; *n* = 5) following the approved protocols outlined in “Participant identification”. To allow possible transmission pathways of the virus to be actively studied, guidelines (as summarized in “Participant identification”) were used to select participants who were most likely to be carrying infectious virus (e.g. high RNA viral load of NP swab when information was available). The mean and median age of the participants were 50.8 and 55 years old, respectively. Further anonymized details of the participants and corresponding sample details are found in the Supplementary Data Tables.

Samples were directly gathered from participants using a custom sampling apparatus which included three main stages in series to capture droplets and bioaerosol particles as an approximate function of their size: macroscopic droplets (Mask Rinse, Stage 1), droplets $\gtrsim$ 10 $\upmu \hbox {m}$ (Rinse of Tubing/Inlet, Stage 2), and bioaerosols $\sim$ 0.3–10 $\upmu \hbox {m}$ (BioSampler, Stage 3). A schematic of the experimental apparatus is shown in Fig. 3 in the “Methods”. The configuration of the sampling setup evolved twice over the project (i.e. Setup A evolved to Setup B, then Setup C) to incorporate learnings, add new equipment, improve the approach and meet the requirements of different environments, as summarized in “Sample apparatus”. The setup utilized with each participant is reflected by the A, B or C in the participant identifiers (e.g. P01A indicates Setup A was used for Participant 1). The BioSampler was used to collect samples from all of the participants (Participants P01A–P17C; *n* = 17), while an Andersen Cascade Impactor (ACI) collected in parallel within Setup A (P01A–P04A; *n* = 4) and the BioSpot-VIVAS (VIVAS) collected in parallel within Setup C (P13C–P17C; *n* = 5). No bioaerosol sampler collected in parallel to the BioSampler within Setup B (P05B–P12B; *n* = 8). A summary of the different configurations is shown in Table 1 of the “Methods”. Further details of these three commercial, bioaerosol samplers are outlined in Section S1 of the Supplementary Information.

As separate samples, participants P01A–P04A and P09B–P12B (*n* = 8) were asked to cough directly into large transparent polyethylene bags (referred to henceforth as a cough bags) and participants P05B–P08B (*n* = 4) were asked to cough directly onto a Petri dish (referred to henceforth as a cough dish). All samples were analyzed ex situ for RNA viral load (by RT-qPCR) and infectivity (by plaque assay).

While the setup changed twice over the study, the NP swab and BioSampler were used to collect similar samples from all 17 study participants. These consistent sampling methods provided the main data for this study’s results and conclusions, and also served as cross-references between the three different test setups. The other bioaerosol samplers used in parallel to the BioSampler or separate sampling methods (i.e. cough bag or cough dish) were used to provide additional data for the same objective and investigate possible improvements to the approach. Further details on participant selection, the sample apparatus, and sample procedure are summarized in the “Methods”.

### Viral concentrations and infectivity

**Figure 1:**
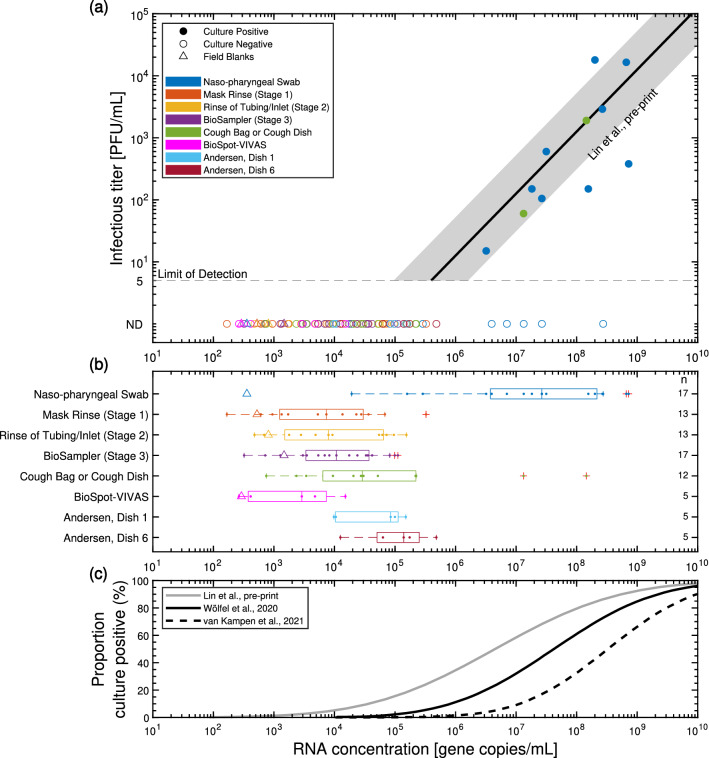
(**a**) Infectious titer determined by viral culturing using plaque assay as a function of gene copies of SARS-CoV-2 RNA (N gene) per mL of sample (comprising viral transport media and respiratory tract fluid) determined by RT-qPCR. Solid or hollow circles were positive or negative by viral culture, respectively (limit of detection: 5 PFU/mL). The dark gray line represents the mean ratio of infectious titer/mL to gene copies/mL for a range of samples using the same viral culture and RT-qPCR methods as in this study^42^. The shaded region shows the 95% confidence interval. (**b**) Gene copies/mL for each sample type as a standard box-and-whisker plot and including all participants in this study. One set of field blanks (denoted by triangles) was gathered from a hospital inpatient not infected with SARS-CoV-2. The small markers depict the results of each individual sample. (**c**) Fraction of samples which showed positive viral culture as a function of gene copies/mL using data from Lin et al.^42^ (33% positive by viral culture) and reproduced from Wölfel et al.^43^ for mostly sputum samples and van Kampen et al.^44^ for samples from the respiratory tract.

Infectious titer determined by viral culturing using plaque assay are shown as a function of gene copies/mL of SARS-CoV-2 RNA (N gene) determined by RT-qPCR in Fig. 1a. The basis for volume (mL) in these measurements of infectious titer (PFU/mL) and RNA concentration (gene copies/mL) consider the total sample volume, consisting of respiratory tract fluid captured and viral transport media (VTM) utilized. The grey line and shaded region is included for context, and shows the mean and 95% confidence interval of the ratio of infectious titer to RNA concentration in clinical samples projected to day zero of symptom onset from a complementary study^42^ in Calgary, Canada. This other study collected $\sim$ 500 samples including NP swabs, sputum and saliva from 75 study participants (4 participants, P01A–P04A, were common with the present study) using the same viral culture and RT-qPCR methods as in this study (as summarized in Supplementary Sect. S2). The infectious titer by plaque assay are positively correlated with gene copies/mL of viral RNA detected by RT-qPCR. In this current work, samples positive for viral culture were NP swabs (53%, 9 of 17, titer: 15–18,000 PFU/mL) and two cough bag samples (25%; 2 of 8, titer: 60 PFU/mL and 1900 PFU/mL). Cough dish samples were culture negative (0 of 4). All samples gathered using the custom sampling apparatus were negative for viral culture (0 of 58; below the limit of detection of 5 PFU/mL), despite sampling air which was directly emitted by participants who carried infectious virus in their respiratory tract as detected by viral culture of their NP swab.

Concentration of SARS-CoV-2 RNA expressed as gene copies/mL are shown in standard box-and-whisker plots for each sample type in Fig. 1b. NP swabs (*n* = 17) had a median of 2.7${\times }10^{7}$ gene copies/mL (Ct: 19.2) and a maximum of 7.2${\times }10^{8}$ gene copies/mL (Ct: 16.2), of which 9 were positive by viral culture of the samples. Note that raw cycle thresholds from RT-qPCR are reported in parentheses following reports of gene copies/mL for a given sample. This conversion from raw cycle thresholds to gene copies/mL is described in Supplementary Sect. S3. The BioSampler (*n* = 17), which samples aerosols of $\sim$ 0.3–10 $\upmu \hbox {m}$ diameter, had a maximum and median RNA concentration of 1.1${\times }10^{5}$ gene copies/mL (Ct: 28.2) and 1.1${\times }10^{4}$ gene copies/mL (Ct: 29.4), respectively. The sample with the maximum RNA concentration was gathered from a participant (P04A) who had an NP swab positive by RT-qPCR (2.0${\times }10^{8}$ gene copies/mL; Ct: 16.9) and by viral culture (18,000 PFU/mL), which was the highest infectious titer of all samples. The cough bag sample from the same participant (P04A) was also positive by RT-qPCR (1.4${\times }10^{8}$ gene copies/mL; Ct: 17.3) and by viral culture (1900 PFU/mL), and corresponded to the highest viral load from all cough bag (*n* = 8) or cough dish (*n* = 4) samples. The maximum RNA viral load of any air sample type from the setups in Fig. 3 was 4.8${\times }10^{5}$ gene copies/mL (Ct: 26.2). This sample was from Dish 6 of the ACI (diameters of bioaerosol particles captured were 0.65–7 $\upmu \hbox {m}$) collected while the participant read aloud a standardized text passage twice (i.e. coughing portion of protocol collected as a different sample). This participant (P02A) had an NP swab positive by RT-qPCR (2.7${\times }10^{8}$ gene copies/mL; Ct: 18.0) and by viral culture (2900 PFU/mL). The cough bag sample from the same participant (P02A) was also positive by RT-qPCR (1.3${\times }10^{7}$ gene copies/mL; Ct: 22.4) and by viral culture (60 PFU/mL). The maximum RNA concentration from the VIVAS (*n* = 5) was 1.5${\times }10^{4}$ gene copies/mL (Ct: 30.4) and was gathered from a participant (P14C) with an NP swab which was positive by RT-qPCR (3.2 ${\times }10^{7}$ gene copies/mL; Ct: 18.8) and by viral culture (600 PFU/mL). The 95% limit of detection (LOD) of the RT-qPCR method was estimated to be $\sim$ 1.3${\times }10^{3}$ gene copies/mL for a typical sample with a 0.14 mL extraction volume (as reported in Supplementary Sect. 2.2). Although the air samples often contained orders of magnitude more SARS-CoV-2 RNA than this LOD, all of these samples were negative for viral culturing by plaque assay.

Based on previous studies, the likelihood of detecting infectious SARS-CoV-2 is positively correlated with the amount of viral RNA detected by RT-qPCR^42^, as shown in Fig. 1c. These similar correlations are from Wölfel et al.^43^, van Kampen et al.^44^ and the complementary study of Lin et al.^42^ previously discussed. Overall, 33% of the plated specimens from the complementary study exhibited some quantity of infectious virus using the same viral culture methods that were implemented in the same laboratory as this study. Lin et al.^42^ also demonstrated that the likelihood of detecting infectious virus by plaque assay increases significantly when RNA is detected in concentrations greater than 1${\times }10^{6}$ gene copies/mL (corresponding to Ct $\sim$ 25). Wölfel et al.^43^ estimated an RNA concentration of 2.5${\times }10^{5}$ RNA copies/mL (95% confidence interval 1.3${\times }10^{4}$–3.2${\times }10^{6}$ RNA copies/mL) has less than a 5% success rate of isolating infectious virus, but the authors did not successfully culture virus samples (mostly sputum samples) for RNA concentrations below 1${\times }10^{6}$ gene copies/mL^17^. For the same < 5% success rate of isolating infectious virus, van Kampen^44^ reported a limit of 4.3${\times }10^{6}$ RNA copies/mL (95% confidence interval 1.7${\times }10^{6}$–8.1${\times }10^{6}$ RNA copies/mL). These thresholds align with other studies which show specimens with Ct > 24 are unlikely to culture^45^, though Ct measurements depend on the RT-qPCR platform used. Despite detecting viral RNA as high as 4.8${\times }10^{5}$ gene copies/mL (Ct: 26.2) in air samples in this work, the complementary study^42^ and other studies^17,43–45^ indicate those samples were unlikely to be plaque assay positive. Furthermore, the five NP swabs shown at the bottom right of Fig. 1a were negative by plaque assay for reasons that were not readily apparent given the increased likelihood of being culture positive as shown in Fig. 1c.

### Viral load by droplet size

**Figure 2:**
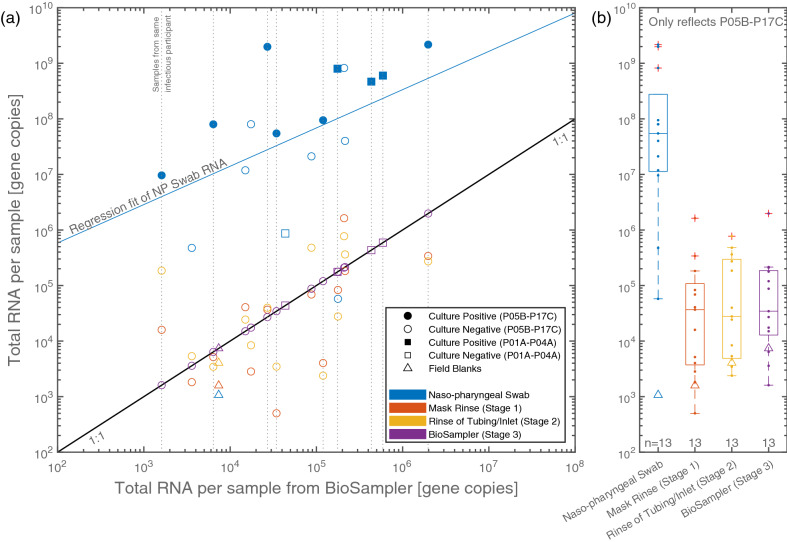
(**a**) Total number of SARS-CoV-2 gene copies per sample from study participants as a function of that found in the air sample gathered concomitantly using the BioSampler (RNA in bioaerosols $\sim$ 0.3–10 $\upmu \hbox {m}$ in diameter; see Supplementary Sect. S3 for details), and (**b**) comparison of total gene copies per sample across the 13 participants (P05B–P17C) for whom NP swabs and samples from Stages 1 to 3 were collected. The small markers depict the results of each individual sample.

Figure 2 shows the total number of SARS-CoV-2 gene copies in samples from study participants as a function of that found in the air sample gathered concomitantly using the BioSampler (i.e. the total RNA in sampled bioaerosols $\sim$ 0.3–10 $\upmu \hbox {m}$ in diameter; see Supplementary Sect. S3 for details). To allow direct comparison between samples, the gene copies (determined by calibration of RT-qPCR) are normalized by the dilution from different volumes of viral transport media and, where applicable, volumetric air flow rates for a given sample type using the equations shown in Supplementary Sect. S3. The SARS-CoV-2 RNA in the NP swabs, macroscopic droplets (Mask Rinse, Stage 1), and droplets $\gtrsim$ 10 $\upmu \hbox {m}$ (Rinse of Tubing/Inlet, Stage 2) are found to correlate with respect to RNA in the bioaerosol samples $\sim$ 0.3–10 $\upmu \hbox {m}$ (BioSampler, Stage 3), as shown in Fig. 2a. This correlation is further supported by the Spearman’s rank correlation coefficient (r), between any two types of samples. Relative to the Stage 3 samples (i.e. BioSampler), the NP swabs (r = 0.50; *n* = 17), Stage 1 (r = 0.68; *n* = 13), and Stage 2 (r = 0.46; *n* = 13) samples each show positive correlations in terms of RNA gene copies.

Generally, more SARS-CoV-2 RNA is detected in samples from a participant if more RNA is detected by their NP swab. However, the samples from Stages 1–3 had roughly 3–4 orders of magnitude fewer gene copies of SARS-CoV-2 RNA than that contained in the corresponding NP swabs. The highest total SARS-CoV-2 RNA gathered by the BioSampler (note: the BioSampler in this case contained 20 mL of VTM rather than the typical 5 mL) was 2.0${\times }10^{6}$ gene copies (Ct: 26.4), an amount higher than the rinses from Stage 1 (3.4${\times }10^{5}$ gene copies; Ct: 27.8) and Stage 2 (2.7${\times }10^{5}$ gene copies; Ct: 28.1) collected from the same participant completing five reps of five forced coughs. This participant (P06B), who participated in the study 2 days after their symptom onset in the community, also had the most SARS-CoV-2 RNA in their NP swab of 2.2${\times }10^{9}$ gene copies corresponding to 7.2${\times }10^{8}$ gene copies/mL (Ct: 16.2) which cultured at 380 PFU/mL.

Total RNA detected in Stages 1, 2 and 3 were comparable in magnitude throughout the range of viral loads reported ($\sim$ 10$^3$–10$^6$ gene copies). To enable a direct comparison, the SARS-CoV-2 RNA results were consolidated for all participants from whom NP swabs and Stages 1–3 were collected (i.e. Participants P05B–P17C, *n* = 13) and shown in Fig. 2b as standard box-and-whisker plots. Participants P05B–P17C nominally performed the same procedure and samples from all stages were gathered (whereas Stages 1 and 2 were not gathered for participants P01A–P04A). From this consolidated dataset, the median number of gene copies were 5.5${\times }10^{7}$, 3.7${\times }10^{4}$, 2.8${\times }10^{4}$ or 3.4${\times }10^{4}$ for RNA samples from the NP Swab, Stage 1, Stage 2, or Stage 3, respectively.

Malik et al.^32^ detected 3–4 orders of magnitude less RNA in exhaled breath (EB) than the same participants’ oro-nasopharyngeal (ONP) swab samples (mean number of gene copies of 7.97${\times }10^{6}$ and 2.47${\times }10^{3}$ for ONP swab and EB samples, respectively), however, a correlation was not established. In this current work, the amount of viral RNA emitted in droplets or bioaerosols were comparable to one another, each positively correlated with viral RNA in participants’ NP swabs, and were found to be 3 to 4 orders of magnitude lower than that of NP swabs in this study.

## Discussion

Results from RT-qPCR indicate SARS-CoV-2 RNA was present in samples of air emitted by participants (with RNA concentrations up to 4.8${\times }10^{5}$ gene copies/mL, Ct: 26.2, of sample from bioaerosol particles between 0.65 to 7 $\upmu \hbox {m}$), including in bioaerosols smaller than 10 $\upmu \hbox {m}$ in diameter. However, all attempts to culture the virus from samples of droplets and bioaerosols from the custom sampling apparatus were negative (0 of 58; limit of detection, 5 PFU/mL) despite sampling air which was directly expired by participants who carried infectious virus as detected by viral culture of their NP swab (53%, 9 of 17, Titer: 15 PFU/mL–18,000 PFU/mL). Two samples for which participants directly coughed into large transparent polyetheylene bags were positive by viral culture (25%; 2 of 8, Titer: 60 PFU/mL and 1900 PFU/mL). While the size of the droplets or bioaerosols which transferred the virus to the bag are unknown, most of the cough bag samples likely contained at least some macroscopic droplets as they were visible to the naked eye on the sides of the bag. Despite detecting viral RNA as high as 4.8${\times }10^{5}$ gene copies/mL (Ct: 26.2 ) in air samples in this work, the complementary study^42^ and other studies^17,43–45^ indicate those samples were unlikely to be plaque assay positive. We did not detect infectious virus in air samples possibly due to insufficient quantity of viral material, limitations of the sampling and analysis techniques^12,46^, challenges of collecting from the most infectious individuals, or the absence of infectious virus in the expiratory air sampled during this study.

In this study, samples were typically gathered within 0 to 5 days of the participant’s first positive NP swab as detected by Alberta Provincial Laboratories COVID-19 testing and on average within 5 days of the participant’s onset of symptoms (range of 0 to 11 days). NP swabs gathered for this study (*n* = 17) were more likely to contain infectious virus if the time from the date of first positive OP or NP swab (as detected by NATs) to the date of sampling was 1–3 days (100%, 7 of 7) rather than 4-5 days (0 of 7). Two NP swabs positive for viral culture were from the same participant (P16C and P17C) at 63 and 69 days from the date of first positive NP swab to the date of sampling. This participant was severely immunocompromised due to being treated with an immunosuppressant medication that depletes B cells. It is known that severe immunodeficiency (e.g. advanced HIV, transplantation, B cell immunodeficiency, hematological malignancy) can prolong viable viral shedding for months^47–50^. For Participant P03A, their first NP swab was gathered concomitantly (i.e. 0 days) with bioaerosol sampling and that NP swab was positive by PCR, but negative by viral culture. The likelihood of maintaining replication- and infection-competent samples increases when samples are gathered early in the course of disease^17,43–45^. Participants in this study were selected based on community NP swab results or were hospital inpatients at the time of sampling. Therefore, most participants in this study had symptoms at the time of sampling (82%, 14 of 17) and during the course of their infection (94%, 16 of 17). Despite not identifying infectious virus in bioaerosols emitted during natural expiratory activities (i.e. coughing, talking and breathing), the representativeness of our findings should be considered. For example, a person possibly earlier in the course of their infection or with different levels of symptoms than participants sampled in this study may have a different outcome. Selection of participants based on recent onset of symptoms or first positive NP swab presents bias in participant selection. However, ambiguity for viral load of respiratory samples (i.e. NP swabs which are a surrogate of the respiratory secretions at the time of air sampling) is avoided by reporting culture and RT-qPCR results for NP swabs gathered concomitantl y with bioaerosol samples.

While this study went to great lengths to identify study participants with the highest viral load (as summarized in “Participant identification”), it was still challenging to identify and enroll such participants in the study. For example, significant time (often several days) commonly elapsed for a potential participant to develop symptoms and recognize the need for testing, for a test to be completed and analyzed, and for the viral load results to be provided for the study’s consideration. Prior research has shown that viral load likely peaks in the first week symptom onset^17^ and this aspect is discussed further in “Participant identification”. Anecdotally, differences in such elapsed times may have contributed to community participants in this study having a higher maximum (18,000 vs 600 PFU/mL) and median (1640 vs 150 PFU/mL) titer compared with hospital inpatients for their NP swabs. Furthermore, potential participants with high viral loads (as identified by community NP swabs) often mentioned experiencing more severe symptoms, and many cited this reason for declining to participate in the study. These external aspects limited the number of potential participants that met the guidelines for participant identification (as outlined in “Participant identification”) and contributed to the smaller sample size of this study (*n* = 17 participants).

The VIVAS was used for hospital inpatients rather than in the community in part due to its size and the time required to reach internal temperatures required for sampling. Of the participants for whom VIVAS samples were gathered (*n* = 5), the participant (P14C) with the highest viral load in their NP swab by RT-qPCR (3.2 ${\times }10^{7}$ gene copies/mL; Ct: 18.8) and viral culture (600 PFU/mL) produced the maximum RNA concentration of all VIVAS samples (1.5 ${\times }10^{4}$ gene copies/mL; Ct: 30.4). However, due to illness, that participant (a) read aloud only half of a standardized text passage, (b) performed 5 forced coughs in total and (c) the mask was held within 3 cm of their face, rather than directly on their face due to their comfort level. This example highlights that, along with possible limitations in sampling methodologies, there were practical challenges in identifying (and sampling from) the most suitable candidates in both community and inpatient settings.

Although NP swabs were successfully cultured at high RNA viral loads in this study, the amount of viral RNA emitted in droplets or bioaerosols from the same participants were found to be 3 orders of magnitude lower on average and none of those samples were culture positive. The RNA detected in samples of emitted droplets and bioaerosols was positively correlated with RNA detected in NP swabs. It is known from previous work that RNA viral load is positively correlated with detection of infectious virus in other types of respiratory specimens (e.g. saliva and sputum), where it is unlikely to detect infectious virus for RNA viral loads < 1 ${\times }10^{6}$ gene copies/mL^17,42^.

Therefore, sampling emitted air from persons with higher viral loads in NP swabs than those who participated in this study or modifying the sample procedures may yield RNA viral loads in emitted air samples > 1 ${\times }10^{6}$ gene copies/mL and may have a higher likelihood of detecting infectious virus. For example, a larger sample size, further improving the methods of identification/mobilization to maximize the proportion of highly-infectious participants, revising the sample collection protocol or further optimizing sampler performance^51^ may increase the likelihood of detecting infectious virus. It is also possible that no infectious virus is present in expiratory specimens of this type, even from highly-infected persons. One possible explanation for this outcome is the viral decay of the SARS-CoV-2 in an aerosol^52–54^. However, the referenced studies of viral decay are limited to laboratory-generated aerosols, which highlights a main challenge of representing and characterizing pathogen-laden bioaerosols. Regardless, this work demonstrates it is highly challenging to retrieve droplets and bioaerosols with viral material greater than > 1 ${\times }10^{6}$ gene copies/mL (e.g. 0 of 58 size-segregated samples), a minimum amount of viral material for which infectious SARS-CoV-2 is typically detected in other respiratory specimens.

While our study is unique in its contribution towards exploring the emission potential for the aerosol transmission pathway, its results must be cumulatively considered with the other aspects of the transmission pathway (such as viral persistence, duration of exposure, dose-response and host defenses) to comment on transmission risks^12–14,55^. By identifying highly-infectious individuals (maximum of 18,000 PFU/mL in NP), we retrieved higher numbers of SARS-CoV-2 RNA gene copies in bioaerosol samples (maximum of 4.8 ${\times }10^{5}$ gene copies/mL and minimum cycle threshold of 26.2) relative to other studies. This outcome highlights improvements over previous methodologies for participant selection, sampling and/or retrieval of high viral emissions, and supports the further implementation of this novel methodology with a larger cohort of study participants.

## Methods

### Participant identification

In this study, potential participants were identified from persons infected with SARS-CoV-2 and likely to be carrying infectious virus. SARS-CoV-2 is often cultured from samples of respiratory tract fluid typically within the first week of symptom onset^17,44,45^. Studies have shown the difficulty of culturing SARS-CoV-2 beyond 8–9 days from symptom onset despite persistently high loads of viral RNA^17,43^. One study showed the probability of successfully culturing SARS-CoV-2 peaked on day 3 from symptom onset and decreased thereafter^45^, whereas a review of 43 studies showed the mean duration of viral shedding of SARS-CoV-2 RNA is 17 days^17^. Therefore, the following guidelines were used to select participants that were most likely to be carrying infectious virus, and thus, allow possible transmission pathways to be actively studied:

1. Recent OP or NP swab that was positive for SARS-CoV-2 RNA (typically within 5 days) as determined by NATs; where available, the cycle threshold (Ct) of this swab was used as secondary indicator, with low Ct values (typically Ct < 20 targeting E gene) being a target given these values indicate high viral shedding;
2. Recent onset of COVID symptoms (ideally within 6 days); and,
3. Other relevant information where available, such as date of likely exposure to SARS-CoV-2 or a recent negative NP swab followed closely by a positive NP swab.

Potential participants who met these guidelines were identified in consultation with Infection Prevention and Control (IPC) site leads at hospitals within the Edmonton Zone, Alberta Public Health and the Alberta Provincial Laboratory following the approved protocols outlined in Research Ethics Board (REB) #Pro00103798. Data for participants P01A–P04A were gathered according to protocols approved by the University of Calgary Conjoint Research Ethics Board (REB20-0444) as outlined in Lin et al.^42^. All methods were performed in accordance with the relevant guidelines and regulations of the approving institutions and in accordance with the Declaration of Helsinki. All of the participants (or designated decision makers on their behalf) provided informed consent to participate in the study. For potential participants in an inpatient setting, the most responsible physician (MRP) was also consulted to assess suitability and possible medical concerns before inviting the patient to participate in the study.

While these guidelines helped identify study participants, a naso-pharyngeal (NP) swab (Flexible Mini-tip FloqSwabs; Copan) was also collected from each participant immediately prior to collecting their emitted air samples. This NP swab was analyzed using both RT-qPCR and viral culturing by plaque assay (as described in “Virological analyses”) to minimize assumptions regarding the participant’s viral load and infectivity of their respiratory tract, and is considered to be a surrogate of the respiratory secretions at the time of air sampling. In one select instance (Participant P14C), an oro-pharyngeal swab (OP) was performed instead of a NP swab due to the participant’s preference. Since NP swabs were typical, NP refers to either NP or OP swab when referring to data in this study.

### Sample apparatus

**Figure 3:**
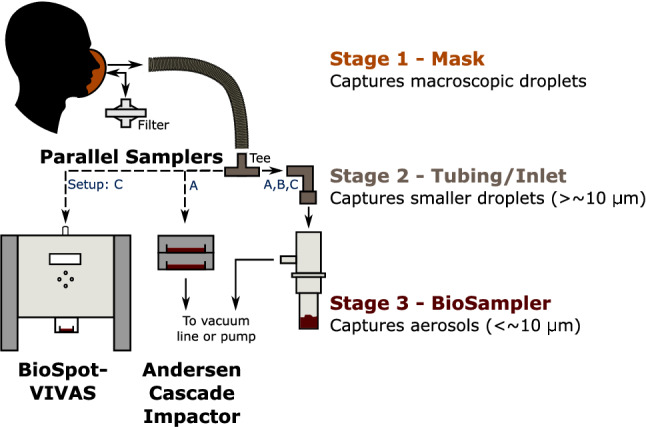
Schematic of experimental setup used to sample bioaerosol that was presumably virus-laden. The evolution of the setup over the duration of the project is summarized in Table 1.

Study participants were asked to breathe, speak and cough into a custom sampling apparatus (consisting of single-use, medical-grade anaesthesia mask and tubing components from a medical breathing system) placed directly over their nose and mouth and the bioaerosols they emitted were continuously sampled from air in the mask by bioaerosol samplers for virological analyses as shown in Fig. 3. A standardized protocol of breathing, speaking and coughing was followed by participants to compare possible viral loads emitted from participants relative to the viral loads of their NP swabs. A vacuum line or pump sampled a flow of expired air at 12.5 L/min into a BioSampler^®^ (SKC Inc., Eighty Four, PA, USA) and another sampler often collected in parallel, specifically the Andersen Cascade Impactor (ACI; Tisch Environmental Inc., OH, USA) or the BioSpot-VIVAS™ Bioaerosol sampler (VIVAS; Aerosol Devices Inc., Fort Collins, CO, USA). A tee and bacterial filter from a medical breathing system was installed on the outlet of the medical mask to provide filtered air (air free of any viruses or bacteria) during participant inhalation and vent any excess air flow expired.

The bioaerosol samplers and the sampling system utilize the mechanism of inertial impaction: that abrupt changes in flow direction of sampled air will cause droplets and bioaerosol particles larger than a threshold size (which are slow to respond to the abrupt change) to impact onto a surface. Further details of these three commercial, bioaerosol samplers are outlined in Supplementary Sect. S1. Using this approach repeatedly, the apparatus was designed to sample droplets and bioaerosol particles depending on their aerodynamic diameter in three main stages (also shown in Fig. 3):

1. Macroscopic droplets impact onto the mask;
2. The sampling tube (made up of connectors and tubing from a medical breathing system) to the inlet of the BioSampler captures smaller droplets. The inlet of the BioSampler includes a 90° elbow which causes droplets ($\gtrsim$ 10 $\upmu \hbox {m}$ in diameter)^56,57^ to deposit by impaction; and
3. The BioSampler captures bioaerosol particles remaining in the air flow ($\sim$ 0.3–10 $\upmu \hbox {m}$^56,58,59^).

See Supplementary Sect. S4 for further details regarding the collection of bioaerosol particles larger than $\approx$ 10 $\upmu \hbox {m}$ in the inlet of the BioSampler due to impaction.

As summarized in Table 1, the configuration of the sampling setup evolved twice over the project to incorporate learnings, add new equipment, improve the approach and meet the requirements of different environments. Of all participants (*n* = 17), Setup A was used for participants P01A to P04A (*n* = 4) in a community setting (i.e. at participants’ homes in their garages or backyards; August 2020), where in parallel to the BioSampler, two stages of a 6-stage ACI sampled air nominally at 28.3 L/min. Particles greater than 7 $\upmu \hbox {m}$ in diameter are deposited in Dish 1 and particles of diameter between 0.65 $\upmu \hbox {m}$ to 7 $\upmu \hbox {m}$ are deposited in Dish 6 of the ACI. Both the BioSampler and ACI used viral transport media (VTM) as the collection liquid. The VTM was Minimum Essential Medium supplemented with L-glutamine, non-essential amino acids, antibiotics/antimycotics (10,000 units penicillin, 10 mg streptomycin and 25 $\upmu \hbox {g}$ amphotericin B per mL), sodium pyruvate, and 2% heat inactivated fetal bovine serum. All tissue culture reagents were purchased from Gibco.

Setup B was used for participants P05B to P12B (*n* = 8) in a community setting (i.e. at participants’ homes in their garages or backyards; November–December 2020). Droplets or bioaerosols that deposited on Stages 1 and 2 of Fig. 3 were rinsed with 3 or 5 mL of VTM immediately after sample collection. Furthermore, to minimize undesired particle losses during transport to the BioSampler, the tubing length between the mask and BioSampler (i.e. Stage 2) was also minimized. This new arrangement was impractical for sampling with the ACI in parallel given its weight and size. Neglecting differences in particle capture efficiencies, the ACI dilutes each viral sample more than the BioSampler, given its higher sample volumes (27 mL of VTM per Petri dish compared with typically 5 mL of VTM in the BioSampler), it separates the particles over multiple sampling stages, and the minimum aerodynamic diameter of captured particles is larger (minimum of 0.65 $\upmu \hbox {m}$ for the ACI compared with minimum of 0.3 $\upmu \hbox {m}$ for the BioSampler). In Setup B, the BioSampler collection vessels were pre-plated with fresh Vero cells to increase the chances of keeping the virus replication- and infection-competent after collection.

Setup C was used for participants P13C to P17C (*n* = 5) in an inpatient setting (i.e. standard ward room; February–March 2021), where in parallel to the BioSampler, a VIVAS was used. The VIVAS collects aerosols over a wider range of particle sizes (0.005 $\upmu \hbox {m}$ to >10 $\upmu \hbox {m}$) than the BioSampler (0.3 $\upmu \hbox {m}$ to $\sim$ 10 $\upmu \hbox {m}$) and may provide a higher likelihood of preserving viability of viruses in air samples due to its gentle collection method. For example, the VIVAS has been demonstrated to improve collection of viable MS2 virus by a factor of 10–100 over the BioSampler^60^. For this study, the VIVAS Petri dish was filled with 1.75 mL of VTM and pre-plated with fresh Vero cells. Other than the VIVAS, Setup C was similar to Setup B (i.e. pre-plated BioSampler collection vessels and rinsing Stages 1 and 2 of Fig. 3 after collection), with the exception of a longer sampling tube (i.e. 22 mm ISO, smooth-bore tube from a medical breathing system) due to the mobility of inpatients often being reduced and space limitations of hospital rooms.

**Table 1:** Different configurations of experimental setup used to collect droplets and bioaerosols emitted during breathing, speaking and coughing.

Setup	Participants	Stage			Parallel samplers	Rinsing of stages 1 & 2?	Cell-plating^e^ of samplers?	Total sample^b^ flow-rate (L/min)
	(*n*)	1	2^a^	3				
A	P01A–P04A (4)	Mask	ISO Tubing^c^	BioSampler	Andersen	No	No	40.8
B	P05B–P12B (8)	Mask	Min. Tubing^d^	BioSampler	None	Yes	Yes	12.5
C	P13C–P17C (5)	Mask	ISO Tubing	BioSampler	VIVAS	Yes	Yes	20.5

^a^For all three setups, the inlet elbow of the BioSampler was considered as part of Stage 2 (including rinse samples) given its capture of smaller droplets ($\gtrsim$ 10 $\upmu \hbox {m}$).

^b^The combined flow-rates of parallel bioaerosol samplers are reported as nominal flow rates recommended by the manufacturers. Where possible, actual flow rates were measured with a bubble or DryCal flow-meter and reported in the Supplementary Data Tables.

^c^The assembly required to split the sample flow between the bioaerosol samplers consisted of a 22 mm ISO tee to 0.75$^{\prime \prime }$ tubing (4$^{\prime \prime }$ long) to a 0.75$^{\prime \prime }$–1$^{\prime \prime }$ barb adapter to 1$^{\prime \prime }$ tubing (4$^{\prime \prime }$ long) to the ACI’s inlet or to 0.75$^{\prime \prime }$ tubing (4$^{\prime \prime }$ long) to 0.75$^{\prime \prime }$–0.5$^{\prime \prime }$ barb adapter to 0.5$^{\prime \prime }$ tubing (30$^{\prime \prime }$ long) to the BioSampler’s elbow inlet.

^d^Minimal plumbing components were used to adapt the mask assembly to the BioSampler inlet. For P05B–P08B, a 3/4$^{\prime \prime }$ tubing (2$^{\prime \prime }$ long) to 3/4$^{\prime \prime }$–1/2$^{\prime \prime }$ barb adapter to 0.5$^{\prime \prime }$ tubing (2$^{\prime \prime }$ long) was used, while for P09B–P12B a 3D printed adapter (22 mm female ISO tapered to 1/2$^{\prime \prime }$) and 1/2$^{\prime \prime }$ tubing (1/2$^{\prime \prime }$ long) was used.

^e^Trial runs demonstrated that the Vero cells at room temperature in VTM after sampling with the BioSampler were found to be viable for at least 24 h after sampling. For the Biosampler collection vessels or VIVAS Petri dishes with pre-plated cells (i.e. utilized in Setups B and C), HEPES was added to the VTM at a final concentration of 20 mM.

The components of Stages 1 and 2 were discarded after sample collection from each participant to avoid cross-contamination between samples or participants. The BioSampler and ACI were sterilized after collecting air samples from each participant.

### Sample procedure

First, an NP swab was gathered and then the participant placed a portion of the custom sampling apparatus over their nose and mouth. The participant was asked “breathe normally”, and, once comfortable, asked to perform the expiratory activities of reading a 330 word passage of text in English (i.e. the “The Rainbow Passage”^61^) and voluntarily coughing 5 repetitions of 5 produced coughs (25 coughs in total). The text passage was used in recent studies which provide detailed measurements of the size distribution of bioaerosols emitted by “healthy” humans during speech and may be used as reference data^4,62^. During sample collection participants were monitored for spontaneous coughing. Any such occurrence was rare and only a few coughs, and therefore considered insignificant relative to the voluntary cough protocol previously discussed.

In a separate analysis, participants P01A–P04A and P09B–P12B (*n* = 8) were each asked to cough directly into a large, sterile, transparent polyethylene bag held 2–4 cm from their mouth and nose (cough bag) and participants P05B–P08B (*n* = 4) were each asked to cough directly onto a Petri dish placed on a table approximately 6 inches from the participant’s mouth (cough dish) in attempts to replicate previous results^35,42^. Cough bags or dishes were not collected from Participants P13C–P17C to minimize the number of forced coughs since these participants were hospital inpatients. Some participants declined to complete or only partially completed some tasks if too onerous. A detailed summary of the tasks completed by each participant during sampling is included in the Supplementary Data Tables.

Non-plated samples were transported to the Biosafety Level 3 (BSL3) virology laboratory in a cooler held between 2–8 $^\circ$C as recommended by the World Health Organization^63^ and confirmed by a temperature logger. Samples for which the collection vessels were plated were transported at approximately room temperature in a sealed, biohazard-rated container. The time between sampling and virological analyses can affect the infectivity of a sample. However, the NP swabs and all other samples for a given participant were handled following the same protocols and similar times between sampling and processing, and all samples were processed within 24 h after collection. Furthermore, the NP swabs gathered in this study were positive for viral culture in 9 of 17 specimens (53%, titer: 15–18,000 PFU/mL), demonstrating that the transport procedures effectively maintained infectivity until sample processing. Although these aspects may affect the infectivity differently based on the sample type, a study based in Calgary^42^ was able to maintain infectivity of different sample types despite having the additional step of transporting them from Calgary to Edmonton for analysis. This complementary study followed similar transport procedures and used the same virological analyses (completed by the same personnel in the same laboratory) as the current study.

### Virological analyses

Calibration of the RT-qPCR was perf ormed by serial dilution of a standard template to determine the approximate number of gene copies per mL of sample for a given RT-qPCR signal. Please see Supplementary Sect. S3 for detailed information on the volumes used in each sample and equations used to normalize the data reported in the Results. For qPCR, 140 $\upmu \hbox {L}$ of sample was typically extracted using a QIAamp viral RNA minikit (Qiagen). Five microliters of RNA were then analyzed using a Promega Go-Taq One-step RT-qPCR kit and Centers for Disease Control and Prevention nucleocapsid (N-gene) primer set and cycling protocol (IDT)^64^. All the qPCR analyses included control wells containing known quantities of an N-gene DNA target. This calibration procedure permits conversion of Ct values to molecular quantities of target.

Viral cult uring by plaque assay was performed on all samples to determine infectivity. A series of 100 µL aliquots, diluted in modified Eagle’s medium (MEM), were plated in duplicate on Vero cells (ATCC Cat# CCL-81), and cultured for 3 or 4 days in MEM containing 1% carboxymethylcellulose. The plaques were fixed and visualized with formaldehyde and crystal violet. Results from viral culturing by plaque assay are reported in plaque forming units per mL of sample (PFU/mL). As an additional confirmation of possible viral replication, aliquots were gathered from culture plates over successive days (up to 2, 3, or 4 days) for 24 of the 92 samples (including NP swabs and field blanks) and RT-qPCR was performed to check for significant increases in RNA concentration over incubation time.

In all samples, the volume of respiratory tract fluid gathered is unknown. For an NP swab, the volume sampled is roughly on the order of 10–100 $\upmu \hbox {L}$, however the volume collected and released into solution will vary^65^. It is expected that the volume of fluid collected in air samples is significantly lower^66^. Both virus titer and RNA concentrations are reported per mL of sample (comprising VTM and respiratory tract fluid), where the volume of respiratory secretion/fluid is assumed to be negligible c ompared to the volume of VTM used (minimum 3 mL).

A detailed description of analytical methods for virological analysis is reported in the Supplementary Sect. 2. Th ese methods were completed under the authorization of the University of Alberta’s Human Research Ethics Board (Pro00099761) and Office of Environmental Health and Safety (RES0052249).

### Statistical information

Sample sizes (*n*) are reported for all statistics calculated, such as shown in Figs. 1b, 2b and Table 1. The data and subsets of interest are represented using standard box-whisker plots. These plots visualize the median of the data, as well as its first and third quartile, which correspond to the median of the lower and upper half of the data, respectively. The difference between the third and first quartile is defined as the interquartile range (IQR) and provides insights into the variation of the data. The whiskers of the plots correspond to 1.5 times the IQR and data that falls outside the whiskers are identified as outliers. If the data is normally distributed, approximately 99.3% of the data falls within the limits of the whiskers.

Spearman’s rank correlation coefficient is also implemented to assess if two parameters from the dataset are related by a monotonic function. As the coefficient approaches − 1 or 1, the relationship between the two parameters approaches a perfectly decreasing or increasing monotonic function, respectively. The sample size (*n*) are also reported for this statistical method, such as in “Viral load by droplet size”.

The 95% limit of detection (LOD) of the RT-qPCR method was estimated to be $\sim$ 1.3 ${\times }10^{3}$ gene copies/mL for a typical sample with a 0.14 mL extraction volume (as reported in Supplementary Sect. 2.2). For PCR measurements below this LOD, the raw measurement was used, rather than translating them to a fixed value (such as done by Leung et al.^67^). Either approach for PCR measurements below the LOD results in values that may not reflect the true quantity of gene copies. However, most of the PCR measurements in this current study are above the LOD. Therefore, translating the PCR measurements below this LOD to a fixed value does not affect the median and third quartile of the box-whisker plots. The effect of this translation on the Spearman’s rank correlation coefficient was also investigated. If these values are shifted to 2 gene copies/mL, the Spearman’s rank correlation coefficients reported in “Viral load by droplet size” remain constant or increase slightly. Therefore, the coefficients reported are a more conservative approach for assessing correlation.

## Supplementary Information

Supplementary Information 1.

Supplementary Information 2.

## Data Availability

All data generated and analysed during this study are included in the Article, Supplementary Information and Supplementary Data Tables.

## References

[1] World Health Organization. Infection prevention and control of epidemic- and pandemic-prone acute respiratory infections in health care. https://www.who.int/publications/i/item/infection-prevention-and-control-of-epidemic-and-pandemic-prone-acute-respiratory-infections-in-health-care (2014). 24983124

[2] World Health Organization. Transmission of SARS-CoV-2: Implications for infection prevention precautions: Scientific brief, 9 July 2020. https://www.who.int/publications/i/item/modes-of-transmission-of-virus-causing-covid-19-implications-for-ipc-precaution-recommendations (2020). (accessed June 2021).

[3] Centers for Disease Control and Prevention (CDC). National Center for Immunization and Respiratory Diseases (NCIRD), Division of Viral Diseases. Scientific Brief: SARS-CoV-2 Transmission. https://www.cdc.gov/coronavirus/2019-ncov/science/science-briefs/sars-cov-2-transmission.html (2021). (accessed June 2021). 34009775

[4] Asadi S (2019). Aerosol emission and superemission during human speech increase with voice loudness. Sci. Rep..

[5] Johnson GR (2011). Modality of human expired aerosol size distributions. J. Aerosol Sci..

[6] Alsved M (2020). Exhaled respiratory particles during singing and talking. Aerosol Sci. Technol..

[7] Gregson FK (2021). Comparing aerosol concentrations and particle size distributions generated by singing, speaking and breathing. Aerosol Sci. Technol..

[8] Duguid JP (1946). The size and the duration of air-carriage of respiratory droplets and droplet-nuclei. J. Hyg..

[9] Bourouiba L, Dehandschoewercker E, Bush JW (2014). Violent expiratory events: On coughing and sneezing. J. Fluid Mech..

[10] Wells WF (1934). On air-borne infection: Study II. Droplets and droplet nuclei. Am. J. Epidemiol..

[11] Hinds, W. C. *Aerosol Technology: Properties, Behavior, and Measurement of Airborne Particles*, 2 ed (Wiley, 1999).

[12] Zhang XS, Duchaine C (2020). SARS-CoV-2 and health care worker protection in low-risk settings: A review of modes of transmission and a novel airborne model involving inhalable particles. Clin. Microbiol. Rev..

[13] Leung NH (2021). Transmissibility and transmission of respiratory viruses. Nat. Rev. Microbiol..

[14] Klompas M, Baker MA, Rhee C (2020). Airborne transmission of SARS-CoV-2: Theoretical considerations and available evidence. JAMA.

[15] Liu Y (2020). Aerodynamic analysis of SARS-CoV-2 in two Wuhan hospitals. Nature.

[16] Chia PY (2020). Detection of air and surface contamination by SARS-CoV-2 in hospital rooms of infected patients. Nat. Commun..

[17] Cevik M (2021). SARS-CoV-2, SARS-CoV, and MERS-CoV viral load dynamics, duration of viral shedding, and infectiousness: A systematic review and meta-analysis. Lancet Microbe.

[18] Zhou J (2021). Investigating Severe Acute Respiratory Syndrome Coronavirus 2 (SARS-CoV-2) surface and air contamination in an acute healthcare setting during the peak of the Coronavirus Disease 2019 (COVID-19) pandemic in London. Clin. Infect. Dis..

[19] Santarpia JL (2020). Aerosol and surface contamination of SARS-CoV-2 observed in quarantine and isolation care. Sci. Rep..

[20] Ong SWX (2021). Lack of viable SARS-CoV-2 among PCR-positive air samples from hospital rooms and community isolation facilities. Infect. Control Hosp. Epidemiol..

[21] Robie ER, Abdelgadir A, Binder RA, Gray GC (2021). Live SARS-CoV-2 is difficult to detect in patient aerosols. Influenza Other Respir. Viruses..

[22] Binder RA (2020). Environmental and aerosolized Severe Acute Respiratory Syndrome Coronavirus 2 among hospitalized Coronavirus Disease 2019 patients. J. Infect. Dis..

[23] Hu J (2020). Distribution of airborne SARS-CoV-2 and possible aerosol transmission in Wuhan hospitals, China. Natl. Sci. Rev..

[24] Nissen K (2020). Long-distance airborne dispersal of SARS-CoV-2 in COVID-19 wards. Sci. Rep..

[25] Lednicky JA (2020). Viable SARS-CoV-2 in the air of a hospital room with COVID-19 patients. Int. J. Infect. Dis..

[26] Lednicky JA (2020). Collection of SARS-CoV-2 virus from the air of a clinic within a university student health care center and analyses of the viral genomic sequence. Aerosol Air Qual. Res..

[27] Lednicky JA (2021). Isolation of SARS-CoV-2 from the air in a car driven by a COVID patient with mild illness. Int. J. Infect. Dis..

[28] Ma J (2020). Coronavirus Disease 2019 patients in earlier stages exhaled millions of Severe Acute Respiratory Syndrome Coronavirus 2 per hour. Clin. Infect. Dis..

[29] Zhou, L. *et al.* Breath-, air- and surface-borne SARS-CoV-2 in hospitals. *J. Aerosol Sci.*** 152**, 105693. 10.1016/j.jaerosci.2020.105693 (2021). 10.1016/j.jaerosci.2020.105693 PMC7557302 33078030

[30] Feng, B. *et al.* Multi-route transmission potential of SARS-CoV-2 in healthcare facilities. *J. Hazard. Mater.*** 402**, 123771. 10.1016/j.jhazmat.2020.123771 (2021). 10.1016/j.jhazmat.2020.123771 PMC7446651 33254782

[31] Ryan DJ (2021). Use of exhaled breath condensate (EBC) in the diagnosis of SARS-CoV-2 (COVID-19). Thorax.

[32] Malik M, Kunze A-C, Bahmer T, Herget-Rosenthal S, Kunze T (2021). SARS-CoV-2: Viral loads of exhaled breath and oronasopharyngeal specimens in hospitalized patients with COVID-19. Int. J. Infect. Dis..

[33] Li, Y. H., Fan, Y. Z., Jiang, L. & Wang, H. B. Aerosol and environmental surface monitoring for SARS-CoV-2 RNA in a designated hospital for severe COVID-19 patients. *Epidemiol. Infect.* **148**, e154. 10.1017/S0950268820001570 (2020). 10.1017/S0950268820001570 PMC7371847 32660668

[34] Samaddar, A. *et al.* Viral ribonucleic acid shedding and transmission potential of asymptomatic and paucisymptomatic coronavirus disease 2019 patients. *Open Forum Infect. Dis.* **8**. 10.1093/ofid/ofaa599 (2020). 10.1093/ofid/ofaa599 PMC7798607 33506066

[35] Kim M-C (2020). Effectiveness of surgical, KF94, and N95 respirator masks in blocking SARS-CoV-2: A controlled comparison in 7 patients. Infect. Dis..

[36] Smolinska A (2021). The SARS-CoV-2 viral load in COVID-19 patients is lower on face mask filters than on nasopharyngeal swabs. Sci. Rep..

[37] Sriraman K (2021). Non-invasive adapted N-95 mask sampling captures variation in viral particles expelled by COVID-19 patients: Implications in understanding SARS-CoV-2 transmission. PLoS ONE.

[38] Williams CM (2021). Exhaled SARS-CoV-2 quantified by face-mask sampling in hospitalised patients with COVID-19. J. Infect..

[39] Cheng VCC (2020). Escalating infection control response to the rapidly evolving epidemiology of the Coronavirus Disease 2019 (COVID-19) due to SARS-CoV-2 in Hong Kong. Infect. Control Hosp. Epidemiol..

[40] Cheng VCC (2020). Air and environmental sampling for SARS-CoV-2 around hospitalized patients with Coronavirus Disease 2019 (COVID-19). Infect. Control Hosp. Epidemiol..

[41] Ding Z (2021). Toilets dominate environmental detection of Severe Acute Respiratory Syndrome Coronavirus 2 in a hospital. Sci. Total Environ..

[42] Lin, Y.-C. *et al.* Detection and quantification of infectious severe acute respiratory coronavirus-2 in diverse clinical and environmental samples from infected patients: Evidence to support respiratory droplet, and direct and indirect contact as significant modes of transmission. *medRxiv*. 10.1101/2021.07.08.21259744 (**Pre-print**).

[43] Wölfel R (2020). Virological assessment of hospitalized patients with COVID-2019. Nature.

[44] van Kampen JJ (2021). Duration and key determinants of infectious virus shedding in hospitalized patients with Coronavirus Disease-2019 (COVID-19). Nat. Commun..

[45] Bullard J (2020). Predicting infectious Severe Acute Respiratory Syndrome Coronavirus 2 from diagnostic samples. Clin. Infect. Dis..

[46] Fennelly KP (2020). Particle sizes of infectious aerosols: Implications for infection control. Lancet Respir. Med..

[47] Aydillo T (2020). Shedding of Viable SARS-CoV-2 after immunosuppressive therapy for cancer. N. Engl. J. Med..

[48] Baang JH (2020). Prolonged Severe Acute Respiratory Syndrome Coronavirus 2 replication in an immunocompromised patient. J. Infect. Dis..

[49] Sepulcri C (2021). The longest persistence of viable SARS-CoV-2 with recurrence of viremia and relapsing symptomatic COVID-19 in an immunocompromised patient - a case study. Open Forum Infect. Dis..

[50] Tarhini H (2021). Long-term Severe Acute Respiratory Syndrome Coronavirus 2 (SARS-CoV-2) infectiousness among three immunocompromised patients: From prolonged viral shedding to SARS-CoV-2 superinfection. J. Infect. Dis..

[51] Ratnesar-Shumate S (2021). Comparison of the performance of aerosol sampling devices for measuring infectious SARS-CoV-2 aerosols. Aerosol Sci. Technol..

[52] Oswin, H. P. *et al.* The dynamics of SARS-CoV-2 infectivity with changes in aerosol microenvironment. *medRxiv*. 10.1101/2022.01.08.22268944 (**Pre-print**). 10.1073/pnas.2200109119 PMC9271203 35763573

[53] Dabisch P (2021). The influence of temperature, humidity, and simulated sunlight on the infectivity of SARS-CoV-2 in aerosols. Aerosol Sci. Technol..

[54] van Doremalen N (2020). Aerosol and surface stability of SARS-CoV-2 as compared with SARS-CoV-1. N. Engl. J. Med..

[55] Jones RM, Brosseau LM (2015). Aerosol transmission of infectious disease. J. Occup. Environ. Med..

[56] Pui DH, Romay-Novas F, Liu BY (1987). Experimental study of particle deposition in bends of circular cross section. Aerosol Sci. Technol..

[57] Lindsley WG (2015). Viable Influenza A virus in airborne particles from human coughs. J. Occup. Environ. Hyg..

[58] Willeke K, Lin X, Grinshpun SA (1998). Improved aerosol collection by combined impaction and centrifugal motion. Aerosol Sci. Technol..

[59] Hogan C (2005). Sampling methodologies and dosage assessment techniques for submicrometre and ultrafine virus aerosol particles. J. Appl. Microbiol..

[60] Pan M (2016). Efficient collection of viable virus aerosol through laminar-flow, water-based condensational particle growth. J. Appl. Microbiol..

[61] Fairbanks, G. Pitch. In *Voice and Articulation Drillbook*, chap. 11, 2 ed, 122–134 (Harper & Row, 1960).

[62] Asadi S (2020). Effect of voicing and articulation manner on aerosol particle emission during human speech. PLoS ONE.

[63] World Health Organization. Laboratory testing for Coronavirus Disease (COVID-19) in suspected human cases. Interim guidance, 2 March 2020. https://apps.who.int/iris/handle/10665/331329 (2020). (accessed June 2021).

[64] Centers for Disease Control and Prevention (CDC). CDC 2019-novel coronavirus (2019-nCoV) real-time RT-PCR diagnostic panel, CDC-006-00019. https://www.fda.gov/media/134922/download (2021). (accessed June 2021).

[65] Warnke, P., Warning, L. & Podbielski, A. Some are more equal - a comparative study on swab uptake and release of bacterial suspensions. *PLOS One ***9**, e102215. 10.1371/journal.pone.0102215 (2014). 10.1371/journal.pone.0102215 PMC4092111 25010422

[66] Cheng Y (2021). Face masks effectively limit the probability of SARS-CoV-2 transmission. Science.

[67] Leung NHL (2020). Respiratory virus shedding in exhaled breath and efficacy of face masks. Nat. Med..

