# Supplementary material for: Viral load of SARS-CoV-2 in droplets and bioaerosols directly captured during breathing, speaking and coughing
Source: Sci Rep. 2022 Mar 3;12:3484. doi: 10.1038/s41598-022-07301-5 (PMC8894466; doi:10.1038/s41598-022-07301-5)
Supplement: Supplementary file 1 — Supplementary Information 1. [file 41598_2022_7301_MOESM1_ESM.pdf]

Supplementary Information: Viral load of SARS-CoV-2 in droplets and bioaerosols directly captured during breathing, speaking and coughing

Tyler J. Johnson[1], Robert T. Nishida[2,*], Ashlesha P. Sonpar[3,4], Yi-Chan James Lin[5], Kimberley A. Watson[6,4], Stephanie W. Smith[3,4], John M. Conly[7,4], David H. Evans[5], and Jason S. Olfert[2,*]

[1]Department of Engineering, University of Cambridge, Cambridge, U.K.
[2]Department of Mechanical Engineering, University of Alberta, Edmonton, Canada
[3]Department of Medicine, Division of Infectious Diseases, University of Alberta, Edmonton, Canada
[4]Alberta Health Services, Alberta, Canada
[5]Department of Medical Microbiology & Immunology and Li Ka Shing Institute of Virology, University of Alberta, Edmonton, Canada
[6]Department of Family Medicine, University of Alberta, Edmonton, Canada
[7]Department of Medicine, Microbiology, Immunology & Infectious Diseases, Pathology & Laboratory Medicine, Synder Institute for Chronic Diseases and O'Brien Institute for Public Health, Cumming School of Medicine, University of Calgary, Calgary, Canada
[*]rnishida@ualberta.ca or jolfert@ualberta.ca

S1 Experimental set-up

The bioaerosol samplers and the sampling system utilize the mechanism of inertial impaction: that abrupt changes in flow direction of sampled air will cause droplets and bioaerosol particles larger than a threshold size (which are slow to respond to the abrupt change) to impact onto a surface. This threshold size is described by the aerodynamic diameter of an aerosol particle (d_a), which is equivalent to its geometric diameter if the particle has a significant liquid component (i.e. has a spherical morphology) and an overall density of 1000 kg/m^3. Given this work focuses on particles consisting of predominantly respiratory fluid (which is primarily liquid water), the terms aerodynamic diameter and particle diameter are used interchangeably when discussing the size of aerosol particles.

Particle size ranges reported for stages of the custom sampling apparatus are characterized by d_{50} cut-off diameters. The d_{50} defines the aerodynamic diameter of aerosol particles which have a 50% penetration efficiency through an impaction stage. Some particles larger than the d_{50} will remain in the flow (not impact onto the target surface), and the likelihood that occurs decreases with increasing diameter (as demonstrated in Section S4).

S1.1 BioSampler

The BioSampler accelerates its sample flow using three glass nozzles above a swirling pool of liquid (in this work, 5 mL or 20 mL of viral transport media; VTM) to collect the bioaerosol particles via inertial impaction and centrifugation[1]. It is among the most efficient methods available for sampling and maintaining infectivity of SARS-CoV-2 bioaerosols as demonstrated in a laboratory setting[2].

S1.2 Andersen Cascade Impactor

The Andersen Cascade Impactor (ACI) uses metal plates, each with 400 uniformly spaced and uniformly sized orifices to direct its sample flow onto a liquid-filled Petri dish below each plate and collect the bioaerosol particles by impaction[3]. The advantage of the ACI is that particles are selected by their aerodynamic diameter (depending mainly on the diameter of the orifices) and deposited in a Petri dish by impaction in each stage. In this study, particles greater than 7 μm in diameter are deposited in Dish 1 (d_{50}=7 μm) and particles of diameter between 0.65 μm to 7 μm are deposited in Dish 6 (d_{50}=0.65 μm). Dishes 2 to 5 were not used in this study.

S1.3 BioSpot-VIVAS

The BioSpot-VIVAS (VIVAS) utilizes four temperature controlled zones to generate a supersaturated water vapor environment using distilled water. The air sample is passed through this environment, which causes the water-vapor to condense and grow the droplets/bioaerosol particles for gentle impaction into a Petri dish filled with liquid. The total liquid volume in the Petri

dish fluctuated slightly during sample collection due to the competing mechanisms of droplet collection adding liquid volume and evaporation into the passing air reducing liquid volume. The VIVAS has been demonstrated to improve collection of viable MS2 virus by a factor of 10-100 over the BioSampler[4]. It is noted that in Pan et al.[4] the BioSampler was operated at 8 L/min rather than 12.5 L/min as recommended by the manufacturer. The VIVAS was also used to collect air samples from a hospital room occupied by two persons infected with SARS-CoV-2[5]. These samples showed cytopathic effects (CPE), which is a possible indication of virus viability. However, CPE on cell monolayers can also be produced by common microbial contaminants, such as bacteria and fungi, and thus is not conclusive evidence of virus infectivity.

S2 Virological analyses

S2.1 Cell culture and virus titering

All tissue culture reagents were purchased from Gibco (Waltham, MA). Vero cells (CCL-81; ATCC, Manassas, VA) were maintained in Minimum Essential Medium (MEM) supplemented with 10% fetal bovine serum (FBS), 2 mM L-glutamine, 1 mM sodium Pyuvate, 1x Non Essential Amino Acids (NEAA), and 1x Antibiotic-Antimycotic.

For virus titering, Vero cells were seeded one day before sample collection into 12-well plates at a density of 2E5 cells/well. Samples received were diluted in a 10-fold serial dilution up to 1,000 folds with MEM supplemented with 2 mM L-glutamine, 1 mM sodium pyruvate, 1x Non Essential Amino Acids (NEAA), and 1x Antibiotic-Antimycotic (SF-MEM). 100 µL of each sample and their dilution series were added to duplicated wells of Vero cells. Plates were incubated in a 37°C Incubator with 5% carbon dioxide for 1 hour with rocking. After the 1 hour absorption period, 1 ml of MEM supplemented with 1% carboxymethylcellulose (CMC), 2 mM L-glutamine, 1 mM sodium Pyuvate, 1x NEAA, and 1x Antibiotic-Antimycotic was added to each well. After 3 days of incubation in a 37 °C incubator with 5% carbon dioxide, the plates were fixed 1:1 with a crystal violet/formaldehyde solution containing 0.13% (w/v) crystal violet, 11% formaldehyde (v/v), and 5% ethanol (v/v) for 1 hour. Plates were de-stained in tap water. Plaques were photographed and counted on a LED white-box.

As an additional confirmation of possible viral replication, aliquots were gathered from culture plates over successive days (up to 2, 3, or 4 days) for 24 of the 92 samples (including NP swabs and field blanks), and RT-qPCR was performed to check for significant increases in RNA concentration over incubation time.

S2.2 Quantitative reverse-transcription polymerase chain reaction (RT-qPCR)

Samples were extracted with QIAamp Viral RNA mini kit (Qiagen). Purified viral RNAs were quantified on a Bio-Rad CFX96 real time PCR system with GoTaq 1-step RT-qPCR systems (Promega), Nucleocapsid (N) specific primers and probes (2019-nCoV CDC RUO kit, IDT), and 2019-nCoV CDC RUO plasmid controls (IDT).

Typically, 0.14 mL was extracted from each sample for RT-qPCR. In an effort to improve the sensitivity of the PT-qPCR testing, 0.375 mL was extracted from the samples collected by the BioSampler and cough dish from Participants P05B to P08B. The extracted volume was then eluted into a volume of 60 µL, from which 5 µL was extracted for RT-qPCR analysis.

RT-qPCR measures cycle thresholds (Ct) for SARS-CoV-2 using the N gene target. A calibration was performed so that the cycle threshold for a given sample was directly related to a number of gene copies detected. Calibration of each RT-qPCR system was performed by serial dilution of a standard template to determine the Starting Quantity (SQ), which was the approximate number of gene copies of SARS-CoV-2 (N gene) RNA detected at a given cycle threshold by PCR. This calibration procedure enabled comparison of results of gene copies of SARS-CoV-2 from different RT-qPCR systems.

The 95% limit of detection (LOD) of the RT-qPCR method was estimated using the "Generic qPCR Limit of Detection (LOD) / Limit of Quantification (LOQ) calculator" provided by Merkes et al.[6]. Based on the PCR runs completed for this study, the LOD of the RT-qPCR method was estimated to be 15.58 gene copies. For typical samples analyzed with an extraction volume of 0.14 mL, the LOD is estimated to be $\sim 1.3 \times 10^3$ gene copies/mL. For the eight samples previously mentioned, which were analyzed with an extraction volume of 0.375 mL, the LOD is estimated to be $\sim 5.0 \times 10^2$ gene copies/mL.

S3 Normalization by sample volumes

Samples were gathered and transported in different volumes of viral transport media (VTM) as specified in the Supplementary Data Tables. Specifically, the samples gathered by rinsing a collection surface used 3 or 5 mL of VTM (i.e. of Stage 1, Stage 2, or cough bag). The collection vessel of the Biosampler contained 5 or 20 mL of VTM, and was also plated with Vero cells when used in Setups B and C. The cough dish contained 3 mL of VTM, while each stage of the ACI contained 27 mL of VTM. The Petri dish of the VIVAS contained 1.75 mL of VTM and plated Vero cells. These volumes of VTM (i.e. 1.75 to 27 mL) were likely orders of magnitude greater than the volume of respiratory fluid sampled and larger than those extracted for virological analyses[7]. To compare the number of gene copies of virus in each sample, the following equations are applied to account for concentration changes of the RNA due to volume extraction and elution during the RT-qPCR analysis, as well as varying collection volumes of VTM and air among the different samples.

The number of gene copies/mL of VTM is reported in Fig. 1 of the main text. This figure demonstrates the correlation between results from plaque assay and RT-qPCR, as well as provides insights into the likelihood a given sample will culture based on the quantity of RNA present. The RNA concentration in each sample is given by,

$$\text{RNA Concentration [gene copies/mL]} = \left(\frac{V_{\text{el}}\ [\mu\text{L}]}{V_{\text{ext}}\ [\text{mL}]}\right)\left(\frac{\text{RNA by PCR [gene copies]}}{V_{\text{PCR}}\ [\mu\text{L}]}\right), \tag{S1}$$

a volume of fluid is extracted (V_{ext}; [mL]) from the sample (typically 0.14 mL) for RT-qPCR. The virus from V_{ext}, is eluted into a volume (V_{el}; [µL]) of solution (typically 60 µL), from which a volume is extracted for analysis (V_{PCR}; [µL]) (typically 5 µL). The volumes used for each sample are specified in the Supplementary Data Tables.

S3.1 Non-air samples

Samples for which the flow rate of air sampled does not affect or is not expected to affect the concentration of RNA in the sample (e.g. NP swab, Mask Rinse [Stage 1], and Rinse of Tubing/Inlet [Stage 2]), the following equation is applied to determine the number of gene copies in each sample,

$$\text{RNA [gene copies]} = V_{\text{s}}\ [\text{mL}] \times \text{RNA Concentration [gene copies/mL]}, \tag{S2}$$

where the total sample volume (V_{s}; [mL]) comprises the volume of VTM and volume of respiratory fluid gathered. In all samples, the volume of respiratory fluid gathered is unknown and is assumed to be negligible compared to the volume of VTM used (minimum of 3 mL VTM).

S3.2 Air samples

In addition to adjusting for the volume of VTM, bioaerosol samples (i.e. those gathered using the BioSampler, ACI and VIVAS) are adjusted for the flow rate of air in the sampler (Q_{s}; L/min) relative to a standard volumetric sample air flow rate (Q_{std}; L/min) of 12.5 L/min of air (which is the nominal sample flow rate of the BioSampler). This conversion allows direct comparison of gene copies of SARS-CoV-2 RNA between the bioaerosol samples and is summarized by the following equation,

$$\text{RNA [gene copies]} = V_{\text{s}}\ [\text{mL}] \times \text{RNA Concentration [gene copies/mL]} \left(\frac{Q_{\text{std}}\ [\text{L/min}]}{Q_{\text{s}}\ [\text{L/min}]}\right). \tag{S3}$$

S4 Deposition in BioSampler inlet

The behaviour of aerosol particles in bends of circular cross-section is reasonably well known. This knowledge has been used previously to estimate deposition in the Biosampler's inlet elbow, which is a 90 ° bend of circular cross-section. Based on Tsai & Pui[8], Lindsley et al.[9] claim that particles 10-15 µm and larger in diameter are expected to collect in the Biosampler's inlet elbow. The following explores this claim in detail.

The following empirical fit[10] for deposition efficiency, η_{lam}, is valid for Reynolds number of 1000 (laminar flow), a curvature ratio (i.e. ratio of bend radius to tube radius) of 5.6 and 5.7, and tube internal diameter ranging from 4 to 8.5 mm as it agrees well with both experimental[11] and numerical[8, 12] methods,

$$\eta_{\text{lam}} = \left(1 + \left(\frac{\text{Stk}}{0.171}\right)^{0.452\frac{\text{Stk}}{0.171}+2.242}\right)^{-\frac{2}{\pi}\varphi} \tag{S4}$$

where φ is the bend angle of the elbow (in radians) and Stk is the Stokes number, a ratio of stopping distance of a particle (due to inertia) to a characteristic dimension. In this case, the characteristic diameter is the inner diameter of the elbow, while the stopping distance of a particle is a function of its aerodynamic diameter and the properties of the gas flow.

Particle deposition for turbulent flow in a bend can be expressed independently of the Reynolds number using the following equation[10],

$$\eta_{\text{turb}} = \exp\left(-2.823\ \text{Stk}\ \varphi\right) \tag{S5}$$

which is an empirical fit to data collected by Pui et al.[11] at Reynolds numbers of 6,000 and 10,000. This equation was also previously used to estimate deposition efficiency at the inlet of a bioaerosol sampler with an inlet similar to the BioSampler[13].

In this work, equations (S4) and (S5) following Brockmann[10] were first verified by comparison with Fig. 6-15 of Brockmann[10] in Fig. S1a. Then, the transport efficiency through the inlet of the BioSampler was estimated using the parameters summarized in Table S1 to find Stk as a function of particle diameter, as shown in Fig. S1b. The Cunningham slip correction factor is calculated following Kim et al.[14]. While the 3.75 curvature ratio of the BioSampler inlet falls outside the 5.6 to 5.7 range for which equation S4 has been validated, this sharper curvature likely results in the equation slightly overestimating the particle sizes captured within the elbow. This overestimation is likely small given that varying the curvature ratio of an elbow between 5 to 30 has an insignificant effect on particle sizes collected in an elbow[11].

Finally, the inlet of the BioSampler is treated as an impaction stage and its d_{50} is estimated as 10.3 µm or 9.9 µm for laminar (equation S4) or turbulent flow (equation S5), respectively. Reynolds number at the inlet of the BioSampler is estimated to be 2188. Regardless of whether the flow is laminar or turbulent, a d_{50} of approximately 10 µm is a reasonable estimate for the deposition cut-off of bioaerosol particles in the inlet of the BioSampler in this work, though further experimental validation should be performed.

Table S1. Parameters used to estimate deposition in inlet of BioSampler

BioSampler properties		
	Volumetric flow rate (L/min)	12.5
	Inner tube diameter (mm)	$\sim$8
	Bend angle (radians)	$\pi/2$
	Bend radius (mm)	$\sim$30
	Curvature ratio	$\sim$3.8
Gas properties		
	Air dynamic viscosity (Pa·s)	1.825×10^{-5}
	Gas density of air at 20°C, (kg/m^3)	1.205
	Temperature (K)	293
	Pressure (Pa)	101325
Calculated		
	Reynolds Number, Re	2188
	Est. d_{50} for laminar flow (equation S4) (µm)	10.3
	Est. d_{50} for turbulent flow (equation S5) (µm)	9.9

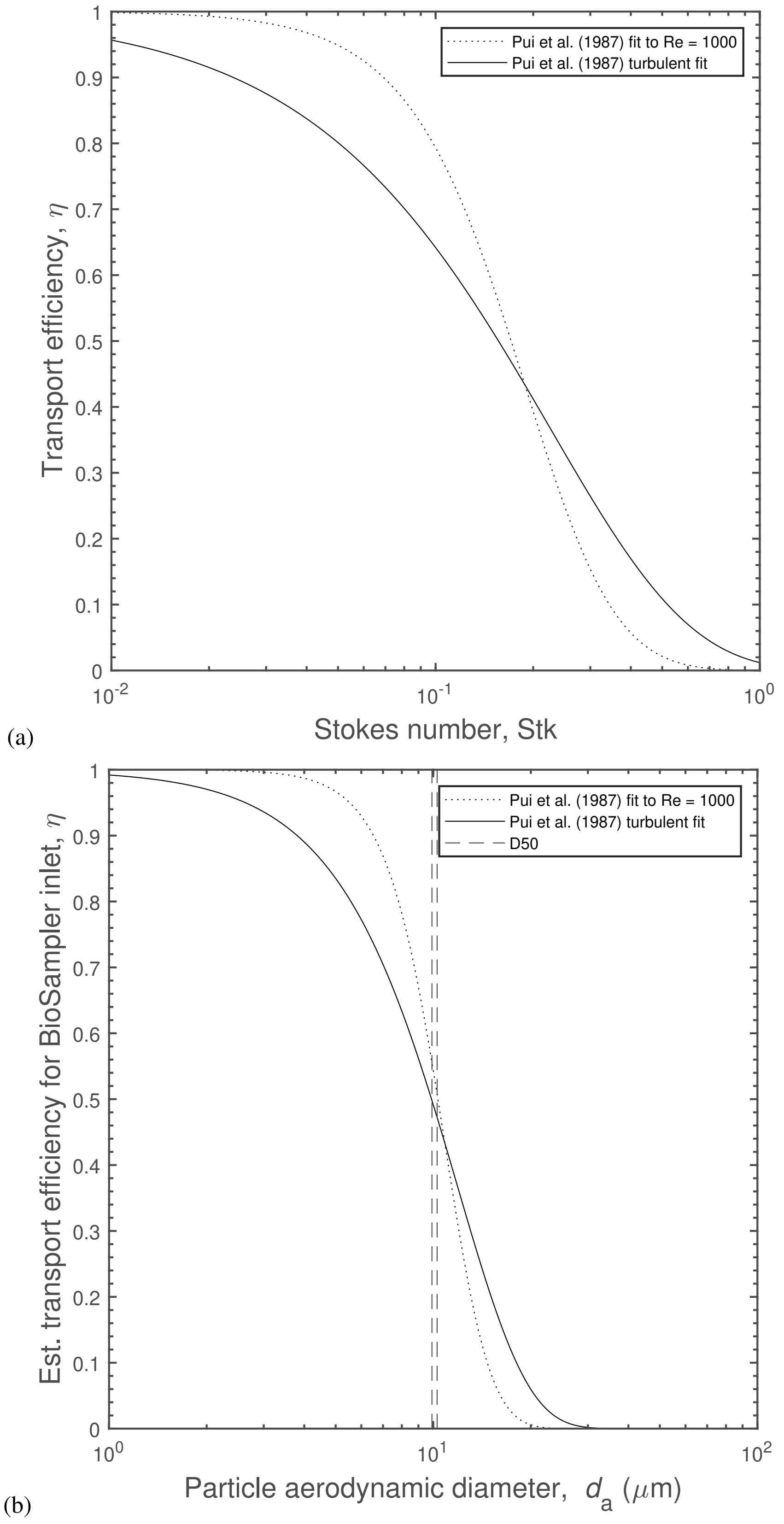

Figure S1. (a) Verification of equations for transport efficiency of particles in an bend as a function of Stokes number by comparison with Fig. 6-15 from Brockmann[10] and (b) estimated transport efficiency of particles through inlet of BioSampler using parameters in Table S1. d_{50} is estimated as 10.3 µm or 9.9 µm for laminar (equation S4) or turbulent flow (equation S5), respectively.

References

1. Willeke, K., Lin, X. & Grinshpun, S. A. Improved aerosol collection by combined impaction and centrifugal motion. *Aerosol Sci. Technol.* **28**, 439–456, DOI: https://doi.org/10.1080/02786829808965536 (1998).
2. Ratnesar-Shumate, S. *et al.* Comparison of the performance of aerosol sampling devices for measuring infectious SARS-CoV-2 aerosols. *Aerosol Sci. Technol.* **55**, 975–986, DOI: https://doi.org/10.1080/02786826.2021.1910137 (2021).
3. Andersen, A. A. New sampler for the collection, sizing, and enumeration of viable airborne particles. *J. Bacteriol.* **76**, 471–484 (1958).
4. Pan, M. *et al.* Efficient collection of viable virus aerosol through laminar-flow, water-based condensational particle growth. *J. Appl. Microbiol.* **120**, 805–815, DOI: https://doi.org/10.1111/jam.13051 (2016).
5. Lednicky, J. A. *et al.* Viable SARS-CoV-2 in the air of a hospital room with COVID-19 patients. *Int. J. Infect. Dis.* **100**, 476–482, DOI: https://doi.org/10.1016/j.ijid.2020.09.025 (2020).
6. Merkes, C. M. *et al.* Generic qPCR Limit of Detection (LOD) / Limit of Quantification (LOQ) calculator. R Script. Available at https://github.com/cmerkes/qPCR_LOD_Calc, DOI: https://doi.org/10.5066/P9GT00GB (2019). Date accessed: 2021-06.
7. Cheng, Y. *et al.* Face masks effectively limit the probability of SARS-CoV-2 transmission. *Science* **372**, 1439–1443, DOI: https://doi.org/10.1126/science.abg6296 (2021).
8. Tsai, C.-J. & Pui, D. Y. H. Numerical study of particle deposition in bends of a circular cross-section-laminar flow regime. *Aerosol Sci. Technol.* **12**, 813–831, DOI: https://doi.org/10.1080/02786829008959395 (1990).
9. Lindsley, W. G. *et al.* Viable Influenza A virus in airborne particles from human coughs. *J. Occup. Environ. Hyg.* **12**, 107–113, DOI: https://doi.org/10.1080/15459624.2014.973113 (2015).
10. Brockmann, J. E. Aerosol transport in sampling lines and inlets. In *Aerosol Measurement*, chap. 6, 68–105, DOI: https://doi.org/10.1002/9781118001684.ch6 (John Wiley & Sons, Ltd, 2011).
11. Pui, D. H., Romay-Novas, F. & Liu, B. Y. Experimental study of particle deposition in bends of circular cross section. *Aerosol Sci. Technol.* **7**, 301–315, DOI: https://doi.org/10.1080/02786828708959166 (1987).
12. Cheng, Y. & Wang, C. Motion of particles in bends of circular pipes. *Atmospheric Environ. (1967)* **15**, 301–306, DOI: https://doi.org/10.1016/0004-6981(81)90032-9 (1981).
13. Grinshpun, S. A., Chang, C.-W., Nevalainen, A. & Willeke, K. Inlet characteristics of bioaerosol samplers. *J. Aerosol Sci.* **25**, 1503–1522, DOI: https://doi.org/10.1016/0021-8502(94)90221-6 (1994).
14. Kim, J. H., Mulholland, G. W., Kukuck, S. R. & Pui, D. Y. Slip correction measurements of certified PSL nanoparticles using a nanometer differential mobility analyzer (nano-DMA) for Knudsen number from 0.5 to 83. *J. Res. Natl. Inst. Standards Technol.* **110**, 31, DOI: https://doi.org/10.6028/jres.110.005 (2005).
